# Enzyme Inhibitory, Physicochemical, and Phytochemical Properties and Botanical Sources of Honey, Bee Pollen, Bee Bread, and Propolis Obtained from the Same Apiary

**DOI:** 10.3390/antiox13121483

**Published:** 2024-12-05

**Authors:** Yusuf Can Gercek, Eda Dagsuyu, Fatma Nur Basturk, Seran Kırkıncı, Nazlıcan Yıldırım, Gamze Kıskanç, Bahar Özmener, Yigit Sabri Unlu, Seda Nur Kalkan, Kadir Boztaş, Gül Cevahir Oz, Refiye Yanardağ, Nesrin Ecem Bayram, Aleksandar Ž. Kostić

**Affiliations:** 1 Department of Biology, Faculty of Science, Istanbul University, Istanbul 34116, Türkiye; kadir.boztas@istanbul.edu.tr (K.B.); cevahirg@istanbul.edu.tr (G.C.O.); 2 Centre for Plant and Herbal Products Research-Development, Istanbul 34116, Türkiye; 3 Department of Chemistry, Faculty of Engineering, Istanbul University-Cerrahpaşa, Istanbul 34320, Türkiye; eda.dagsuyu@iuc.edu.tr (E.D.); yanardag@iuc.edu.tr (R.Y.); 4 Institute of Graduate Studies in Sciences, Istanbul University, Suleymaniye, Istanbul 34116, Türkiye; fnbasturk@istanbul.edu.tr (F.N.B.); serankirkinci@ogr.iu.edu.tr (S.K.); nazlican.yildirim@ogr.iu.edu.tr (N.Y.); gamze.kiskanc@ogr.iu.edu.tr (G.K.); baharozmener@ogr.iu.edu.tr (B.Ö.); yigitsabri.unlu@ogr.iu.edu.tr (Y.S.U.); sedanurk@ogr.iu.edu.tr (S.N.K.); 5 Department of Food Processing, Aydıntepe Vocational College, Bayburt University, Bayburt 69500, Türkiye; nesrinbayram@bayburt.edu.tr; 6 Department of Chemistry and Biochemistry, Faculty of Agriculture, University of Belgrade, 11080 Belgrade, Serbia

**Keywords:** bee products, botanical origin, phenolics, sugars, antioxidant activity, enzyme inhibition

## Abstract

Bee products are an important source of nutrients and bioactive phytochemicals. This study aimed to determine the chemical composition (proximate composition, general phytochemical composition, sugar, and phenolic profiles) of four different products (honey, bee pollen, bee bread, and propolis), obtained from the same apiary, as well as to assess their biological activity through antioxidant and enzyme inhibition assays (α-amylase, α-glucosidase, lipase, AchE, neuraminidase, angiotensin-converting enzyme, urease, trypsin, tyrosinase, carbonic anhydrase, thioredoxin reductase, adenosine deaminase). Clear differences were observed among the samples in terms of both chemical composition and biological activity. The analysis revealed that bee pollen exhibited the highest carbohydrate content (87.9%), while propolis was identified as the richest source of phenolic compounds (14,858.9 mg/kg) among the analyzed samples. Propolis exhibited the highest biological activity in all applied antioxidant assays (CUPRAC, DPPH^•^, and ABTS^•+^) and in most enzyme inhibition assays. Notably, the α-glucosidase inhibition activity of propolis was comparable to that of the reference standard. In addition, honey exhibited remarkable trypsin inhibition, also comparable to the applied standard. These findings highlight the diverse bioactivities of hive products, which could play a key role in promoting health and preventing diseases.

## 1. Introduction

In the food industry, there is a continuous search for solutions to improve food quality, safety and functionality, and at this point, the interest in different bee products such as honey, pollen, and propolis is increasing day by day [1]. Because bee products are preferred by people for many purposes due to their unique bioactive properties, they are considered among the most adulterated food/functional food products.

As a crossroad between plant/animal kingdoms, bee products (honey, bee pollen, bee bread, as well as propolis) attract high attention among consumers, in particular for those oriented towards healthy diets, the use of functional foods, and therapeutics. Honey (BH), the main bee product and the energy source for bees, has been used in the human diet and phytotherapy as early as the Stone Age due to its bioactive compounds associated with beneficial health characteristics [2,3]. Bee pollen (BP) is the main nutrient source for the entire colony, providing essential nutrients (proteins, carbohydrates, lipids such as fatty acids and sterols, vitamins, and minerals), with varying amounts depending on environmental conditions and floral origins [4,5,6,7,8]. Similarly, the basic nutritional requirements of humans include proteins, carbohydrates, lipids, minerals, and vitamins [9]. In this respect, BP is preferred by consumers due to its rich bioactive content [10]. On the other hand, another bee product obtained by fermenting bee pollen in honeycomb cells is called bee bread (BB). It is also nutritionally important due to the presence of a remarkable amount of lipids, proteins, microelements, vitamins, and phenolic compounds [10,11]. The chemical composition of BB may differ in some respects compared to bee pollen [12,13]. Investigations have shown that BB has higher nutrients and phytochemicals bioavailability [14,15]. Apart from these three bee products, which are primarily related to bee nutrition, propolis (BPr) is also a very important hive product. Propolis is collected by bees from different plant sources, such as poplar, pine, palm, resins, and gums, as well as from some leaf buds and mucilage. It presents resinous material found on hive walls, used to protect bees from different parasites, intruders, and diseases [16]. Unlike the previously listed products, BPr is an exclusive source of phytochemicals, with expressed bioactivity and a high application in the human diet and medicine.

However, all these bee products are well-documented sources of bioactive compounds with high efficacy in the treatment of several different diseases and disorders [17]. Bee products are valuable sources of antioxidants that reduce reactive oxygen species (ROS) in cells and eliminate their adverse effects, thanks to their rich phenolic content [18,19,20]. There are studies reporting that BH has many biological and therapeutic properties [21]. However, the biological and therapeutic properties of BP, such as antibacterial, antifungal antimutagenic, antioxidant, and anti-inflammatory activities, have been reported by Pascoal et al. [22] and reviewed by Denisow and Denisow-Pietrzyk [23]. Similarly, in vitro studies of BB have shown antioxidant [24,25], antibacterial [26,27], and anticancer [28,29,30] properties. Finally, there are many studies emphasizing the numerous bioactive properties of BPr [31,32]. The dietary properties, chemical content, and biological activities of bee products depend on many factors, including climate, seasonal variation, soil type, floral diversity, collection methods, storage conditions, and extraction techniques [33,34,35]. Therefore, determining plant origins, chemical content, and biological activity (including antioxidant capacity) is crucial for the standardization of bee products [33].

The use of honey and honey products in the control and treatment of skin; diabetes; asthma; and cardiovascular, neurological, tuberculosis, hepatotoxic, and gastrointestinal diseases has been demonstrated [36]. In addition, diabetes [37] and neuro-inflammatory diseases [38] are among the most spread disorders triggered by the activity of different enzymes in the human body. One of the potential strategies to enhance pharmacotherapy for diabetes and Alzheimer’s disease (AD) is to control the activities of the enzymes associated with the pathophysiology of these diseases. Recent literature suggests that BP can serve as an excellent source of quercetin and its derivatives—a group of compounds well known for their potency in preventing and suppressing neuroinflammation [39].

Apart from this, several enzymes have a significant influence on the development of the influenza virus [40], cardiovascular diseases and infections [41], peptic and gastric ulcers, acute pancreatitis, albinism, epilepsy, glaucoma, gastritis, renal, and pancreatic malignant brain tumors [42,43,44], as well as antioxidant defense system and autoimmune diseases [45,46]. In order to avoid use of synthetic drugs, there is a constant tendency to find natural remedies for all these disorders, including using bee products.

While there has been extensive research on the phytochemical characterization, botanical origin determination, and inhibitory activities of BH, BP, and BB on enzymes, studies focusing on the comparison of bee product samples from the same apiary remain rare. Hence, this study aims to determine the nutritional properties and chemical composition of BH, BP, BPr, and BB samples from the same apiary, as well as to assess their anti-inflammatory potential. In this context, proximate composition, general phytochemical composition, along with detailed sugar and phenolic profiles were determined by using HPLC-RID and LC-MS/MS techniques, respectively. To evaluate the health benefits of BH, BP, BB, and BPr as dietary supplements, this study aimed to examine biological activity of bee product extracts. For this purpose, the antioxidant potential and the inhibitory effects on twelve enzymes were evaluated for BH, BP, BPr, and BB extracts.

## 2. Materials and Methods

### 2.1. Collection of Samples

BH, BP, BB, and BPr samples were collected from the same apiary in the Aydıntepe region of Bayburt, (Türkiye). For each sample group, 100 g of samples were collected from a single beehive. To ensure homogeneity, the samples were mixed prior to analysis. Bee pollen, propolis, and bee bread samples were stored in a freezer at −18 °C, while honey was stored at room temperature until analysis.

### 2.2. Botanical Origin of SAMPLES

The botanical origin of BP and BB samples was determined according to the method proposed by Mayda et al. [24]. The botanical origin of the BH sample was determined following the methods proposed by Sorkun [47] and Louveaux et al. [48].

### 2.3. Preparation of Sample Extracts

For extract preparation, 1 g of BB, BP, and BPr sample was extracted with 10 mL of 70% ethanol (LC Grade), while 1 g of BH was extracted with 10 mL of ultrapure water. The extraction was performed using an ultrasonic water bath at 56 KHz (Kudos, SK22110 model, Shanghai, China). Total extraction time was adjusted at 50 min. After extraction, the samples were centrifuged at 10,000 rpm for 10 min (Beckman Coulter Allegra X-30R Centrifuge (Beckman Coulter, Brea, CA, USA), C0650 rotor), and the supernatant was stored at −80 °C until use in spectrophotometric phytochemical analyses and phenolic quantification by LC-MS/MS.

### 2.4. Proximate Composition and Color Analysis of Samples

Total protein content was determined using the Bradford protein determination microplate assay [49,50]. Prior to the Bradford assay, a 500 mg sample was homogenized and hydrolyzed with 30 mL of 0.1 M NaOH in a 3.5% NaCl alkaline solution [51]. Further, 50 µL of alkaline extract was added into the microplate well. A total of 200 µL of Bradford reagent was added, and the mixture was mixed with a pipette. After 15 min of waiting, absorbance was measured at 590 nm. Total lipid content was determined using the Bligh and Dyer assay [52]. One gram of sample was weighed, 4 mL of methanol and 2 mL of chloroform were added, and the mixture was vortexed for 2 min. Further, 3.6 mL of distilled water was added to the mixture, and it was vortexed for 2 min. Centrifugation at 2000 rpm for 10 min was performed to achieve phase separation, and the lower phase was transferred to a glass vial. In the second extraction step, 400 μL methanol and 3.6 mL chloroform were added to the mixture. After vortexing for 2 min, centrifugation was performed at 2000 rpm for 10 min. The lower phases were combined and evaporated at 104 °C. Ash and moisture content of the samples were determined according to AOAC methods AOAC 920.181-1920 and AOAC 930.04-1930, respectively [53]. Total carbohydrate content was calculated using the previously described method [54], by the formula: carbohydrates = 100 − (g fat + g protein + g ash). The results for ash, total protein, carbohydrate, and lipid content were expressed as percentages. Energy was calculated using the Atwater method [55], which estimates total energy from the protein, lipid, and carbohydrate content of foods as

Energy (kcal) = [4 kcal/gram × g protein) + (4 kcal/gram × g carbohydrate) + (4 kcal/gram × g lipid)].

All results are expressed on a dry weight (DW) basis. Color values of the samples were obtained using an NH300 model colorimeter (3 nh, Guangzhou, China) based on the CIELAB(Lab) color system. Prior to analysis, the instrument was calibrated with a white ceramic plate. BH, BP, BPr, and BB samples were placed on a white plate. Lightness (L*), hue (H*) and chromaticity parameters red/green value (a*) and blue/yellow value (b*) were recorded. For each sample, color was measured at three different positions.

### 2.5. Phytochemical Analysis of Samples

#### 2.5.1. Total Phenolic, Flavonoid, Anthocyanin, and Proanthocyanidin Contents

The phytochemical analysis includes the determination of total phenolic, total flavonoid, total anthocyanin, total proanthocyanidin, and total carotenoid contents. Total phenolic content of the extracts was measured using the Folin–Ciocâlteu microplate method [56], with results expressed as gallic acid equivalents (mg GAE/g) based on fresh weight (FW) of sample. For this purpose, 50 µL of the extract was transferred to the microplate well. Then, 50 µL Folin–Ciocâlteu reagent and 100 µL 0.35 M NaOH were added. After pipetting, the mixture was kept for 3 min at room temperature, and absorbance was measured at 760 nm. Total flavonoid content of extracts was measured using the standard AlCl_3_ spectrophotometric method, with the modifications described in the literature [57,58]. For this purpose, 125 μL of sample extract was transferred to the test tube, and 625 μL of dH_2_O and 37.5 μL of NaNO_2_ (5%, *w*/*v*) were added. After vortexing, the mixture was kept at room temperature for 6 min. Then, 75 μL AlCl_3_ (10%, *w*/*v*) was added to the mixture, and the tubes were vortexed and kept in the dark for 5 min. In the last step, 250 μL of 1 M NaOH and 138 μL of distilled water were added, and the mixture was allowed to stand for 20 min. Absorbance was recorded at 510 nm, and the results were expressed as quercetin equivalents per gram of the sample (mg QE/g FW). The total anthocyanin content of the extracts was determined using the pH differential method [59]. For this purpose, 8 mL of pH = 1 buffer solution (0.025 M KCl) and pH = 4.5 buffer solution (0.4 M sodium acetate) were added separately to 2 mL of extract. The mixture was vortexed and left to wait for 15 min at room temperature. After waiting, the absorbance of the reaction was measured at 520 and 700 nm. Cyanidin-3-glucoside was used as the standard in this experiment. Total proanthocyanidin content in the extracts was determined following the method by Broadhurst and Jones [60]. After that, 50 µL of sample extract was transferred into test tubes. Then, 4% vanillin solution (*w*/*v*) dissolved in methanol and 30% HCl (*v*/*v*) were added to the test tube. After vortexing, the mixture was kept at 37 °C for 15 min. Absorbance of mixture recorded at 500 nm, and results were expressed as catechin equivalents (mg CE/g FW). Total carotenoid content was quantified according to Kostić et al. [58]. For this purpose, 500 mg of the sample was weighed and transferred into test tubes. After adding 5 mL of 80% (*v*/*v*) acetone to the sample, the samples were thoroughly mixed with the help of a vortex. The mixture was kept in the dark at room temperature for 12 h with continuous shaking at 200 rpm. After 12 h, another 5 mL of 80% acetone was added to the samples, and the mixture was kept in the dark, at room temperature, with shaking at 200 rpm for 12 h. After 24 h, the samples were centrifuged at 10,000 rpm for 10 min, and 1 mL of the supernatant was transferred to a spectrophotometer cuvette, with absorbance measured at 450 nm. β-Carotene was used as the standard solution, and the absorbance of the mixture was measured at 450 nm, with the results presented as µg/g FW.

#### 2.5.2. Total Antioxidant Capacity Assays

CUPRAC, DPPH^•^, and ABTS^•+^ assays were used to determine the antioxidant capacity of the samples. The CUPRAC assay was performed following the method of Apak et al. [61]. For this purpose, 1 mL of 10^−2^ M CuCl_2_, 1 mL of 7.5 × 10^−3^ M Neocuproine dissolved in 96% ethanol (*w*/*v*), and 1 mL of 1 M ammonium acetate were transferred to test tubes. After that, 1.1 mL of sample was added to the mixture, and the mixture was vortexed and kept at room temperature for 30 min. Absorbance of the mixture was measured at 450 nm. The DPPH^•^ assay was performed by modifying the sample volume in the method of Bobo-García et al. [62]. The mixture of 50 µL of the sample and 150 µL of DPPH^•^ solution (150 µmol L^−1^) in methanol was transferred to the microplate well. After pipetting, the mixture was kept in the dark for 20 min. Absorbance of the mixture was measured at 510 nm. The ABTS radical cation assay was carried out according to Re et al. [63]. For this purpose, 1 mL of ABTS radical cation diluted with ethanol at an absorbance of 0.650–0.700, 1 mL of sample, and 3 mL of ethanol were transferred to test tubes. The mixture was vortexed and held at room temperature for 6 min. Absorbance of the reactant was measured at 734 nm. Trolox was used as the standard for CUPRAC, DPPH^•^, and ABTS^•+^ assays, and the antioxidant capacity of the samples was expressed as Trolox equivalents (mg TQ/g) and presented based on the FW of the samples.

### 2.6. Analysis of Sugars

Sugar extraction was carried out using an ultrasonic water bath. For this procedure, 500 mg of the sample was extracted with 10 mL of dH_2_O. Extraction of sugars was performed at 56 kHz frequency for 50 min. The extract of each sample was transferred to a 15 mL centrifuge tube, 1.5 mL of ultrapure water was added, and the mixture was incubated at 60 °C for 10 min. Then, 0.25 mL of Carrez I solution, 0.25 mL of Carrez II solution, and 1 mL of acetonitrile were added to the mixture. The tubes were gently shaken and kept at room temperature for 1 h, without further mixing. The mixture was then centrifuged at 10,000× *g* for 8 min at 20 °C. Supernatants containing sugars were filtered through a 0.45 µm nylon membrane [64]. Chromatographic analysis of the samples was performed using a RefractoMax520 RI detector (ERC, Thermo Fisher Scientific, Waltham, MA, USA) with the HPLC system (Thermo Fisher Scientific, Waltham, MA, USA), which consisted of a binary pump, autosampler, and column thermostat. Separation was carried out using an HPLC column (Thermo Scientific, Hypersil GOLD Amino, 150 mm × 4.6 mm diameter, 3 µm particle size). The mobile phase consisted of acetonitrile/water (*v*:*v*, 80/20). The column was maintained at 45 °C. The injection volume was 10 µL, and the flow rate was 1.2 mL/min. Detector temperature, response time, and data collection parameters were set at 35 °C, 1 s, and 5 Hz, respectively [65]. Data acquisition and processing were performed using Chromeleon 7.1 (Thermo LC-MS software). Results are expressed as g/100 g of FW of the sample.

### 2.7. Quantification of Phenolics by LC-MS/MS

Phenolic extraction was carried out according to the method described by Zhou et al. [66]. For this purpose, 100 µL of the extract was mixed with 900 µL of extraction solvent (water/methanol/formic acid, *v*:*v*:*v*, 80:19:1). The mixture was then homogenized using a sonicator at 45 °C for 30 min. Following homogenization, the samples were centrifuged at 13,500 rpm for 5 min (Eppendorf 5417R centrifuge (Eppendorf, Hamburg, Germany), F45-30-11 rotor), and the supernatant was stored at −20 °C until further analysis.

LC-MS/MS analysis was performed using a Thermo TSQ Quantis (Thermo Fisher Scientific, Waltham, MA, USA) equipped with an electrospray ionization (ESI) interface. Sample separation was conducted using a Thermo Accucore^TM^ C18 HPLC column (150 mm × 2.1 mm diameter, 2.6 µm particle size). The LC conditions were as follows: autosampler temperature at 4 °C, column temperature at 30 °C, and flow rate of 0.2 mL/min; the mobile phases used were 5 mM ammonium acetate (mobile phase A) and 1% acetic acid in an acetonitrile/methanol (*v*:*v*, 1:1) solution (mobile phase B); the injection volume was set to 5 µL and a 28 min run time. MS conditions were set to spray voltage of 3 kV in both positive and negative modes; sheath gas flow at 54 arb, auxiliary gas flow at 11 arb, sweep gas flow at 1 arb, ion transfer tube temperature at 275 °C, and vaporizer temperature at 400 °C. Data acquisition and processing were performed using Chromeleon 7.1 (Thermo LC-MS software). In this study, 28 phenolic substances (Table S1) were investigated and the obtained results were expressed as mg/kg of fresh weight (FW) of the samples. Quantification and confirming ions for used standards have been presented in Supplementary (Table S1).

### 2.8. Enzyme Inhibition

All inhibitor solutions were prepared by dissolving them in 95% ethanol. To investigate the inhibition activity of the samples, an in vitro α-amylase inhibition assay was performed using a slightly modified method described by Oboh et al. [67]. An in vitro α-glucosidase inhibition assay was performed using a 96-well microwell plate with *p*-nitrophenyl-α-d-glucopyranoside (PNPG, Sigma Ald. (St. Louis, MO, USA) N1377) as the substrate, following a slightly modified method by Tao et al. [68]. Lipase inhibition was measured by monitoring the hydrolysis of *p*-nitrophenyl caprate (*p*-NPC), which releases the yellow chromogen, *p*-nitrophenol [69]. Acetylcholinesterase inhibitory activity was determined by a colorimetric assay based on Ellman’s reaction [70]. Neuraminidase inhibitory activity was determined by using the method of Myers et al. [71]. The angiotensin-converting enzyme (ACE) inhibition assay was performed in microplates, using ((N-[3-(2-furyl) acryloyl]-L-phenylalanyl-glycyl-glycine) (FAPGG) as the substrate, as described by Shalaby et al. [72]. Urease inhibition activity was measured using the indophenol method as per Hanif et al. [73]. Trypsin inhibitory activity was determined following the method by Ribeiro et al., which depends on the hydrolysis of N-α-benzoyl-DL-arginine-*p*-nitroanilide (BAPNA) by trypsin [74]. Tyrosinase inhibition was assessed according to the method by Hwang and Lee [75]. Carbonic anhydrase inhibition was measured based on the reduction of esterase activity using 4-nitrophenyl acetate (4-NPA) as a substrate [76]. Thioredoxin reductase activity was determined using a modified version of the method described by Holmgren [77]. Adenosine deaminase inhibition was performed following the method of Blum and Schwedt [78]. The obtained results are expressed as % of inhibition of examined enzymes as well as IC_50_ values.

### 2.9. Statistical Analysis

The results of general phytochemical, phenolic, and sugar analyses were expressed as a mean ± standard deviation (SD) of the mean of replications for each analyzed extract. Statistical analysis was conducted using the Minitab 19 statistics program (State College, PA, USA). Normal distribution of the data was tested by a Ryan–Joiner normality test. According to the normality test result, it was determined that the data were normally distributed. The significance of the results was evaluated using one-way ANOVA, followed by Tukey’s post hoc test to rank significance. All results are presented as means ± SD, with significance levels set at *p* < 0.05.

## 3. Results

### 3.1. Botanical Origins of Samples

Plant pollens belonging to the families Asteraceae, Boraginaceae, Brassicaceae, Campanulaceae, Fabaceae, Lamiaceae, Malvaceae, Polygonaceae, Ranunculacae, Rosaceae, and Salicaceae were commonly identified in the samples (Table 1). Eight different genera were found in the BH and BP samples, while nine genera were found in the BB samples. The most represented families across all samples were Fabaceae, Asteraceae, and Lamiaceae. Regarding the general distribution of Asteraceae and Fabaceae, these families were secondary distributors across all three different bee products. The total percentages of Fabaceae taxa were 48%, 64.2%, and 61.6% for BH, BP, and BB samples, respectively, while the total percentages of Asteraceae taxa were 19.71%, 17%, and 24% respectively.

### 3.2. Proximate Composition Results

The results for the proximate composition of BB, BH, BP, and BPr are presented in Table 2.

It was observed that carbohydrates were the most predominant nutrients in all examined bee products, ranging from 58.2% (BPr) to 87.9% (BP), followed by lipids (4.2–26.5%) and proteins (0.08–13.2%). All products exhibited low, favorable moisture content, lower than 10%. In addition to its low protein content, BH was characterized by a notably low ash content (0.09%) compared to the other bee products (2.0–3.6%). However, BH (446.5 kcal) and BPr (520.9 kcal) had higher energy values compared to BP and BB (375.3–396.0 kcal).

### 3.3. General Phytochemical Composition

#### 3.3.1. Total Phenolic, Flavonoid, Anthocyanin, Carotenoid, and Proanthocyanidin Contents

The results for the general phytochemical composition, expressed through total phenolic (TPC), total flavonoid (TFC), total proanthocyanidin (TPAC), total carotenoid (TCC), and total anthocyanin (TA) contents in the analyzed bee products are summarized in Table 3.

According to the results, TPC values varied significantly, with the BH sample (0.4 mg/g GAE FW) showing the lowest TPC, and the BPr sample having the highest TPC content (85.3 mg/g GAE/FW). A similar trend was observed for TFC (0.04 mg/g QE/FW (BH) to 44.8 mg/g QE/FW (BPr)), as well as for TCC (2.7–208.7 μg/g FW) and TPAC (0.1–1.5 mg CE/g/FW) parameters. In contrast, TA was not detected in any of the bee products.

#### 3.3.2. Antioxidant Capacity of Bee Bread, Honey, Pollen, and Propolis

The results of the three different antioxidant assays performed on the examined bee products are presented in Table 3. All obtained results align with the general phytochemical composition, with BH showing the lowest antioxidant activity (0.6 mg/g TE FW for CUPRAC, 1.3 mg/g TE FW for DPPH^•^, 3.3 mg/g TE FW for ABTS^•+^). In contrast, BPr exhibited the highest observed antioxidant activity across all three assays (133.8 mg/g TE FW for CUPRAC, 71.3 mg/g TE FW for DPPH^•^, 71.3 mg/g TE FW for ABTS^•+^).

### 3.4. Results of Color Analysis

The color of bee products is a significant physicochemical parameter that contributes to the characterization of the samples. The colorimetric characteristics of the surfaces of the BH, BP, BB, and BPr samples are presented in Table 4. Among the analyzed samples, bee pollen stood out with the highest values for L* (59.3), a* (8.74), b* (28.2), C (29.6), and H (72.8) parameters.

### 3.5. Sugars Profile

To determine the sugar composition, as the predominant nutrients in all bee products, UHPLC-RID analysis was conducted. The results are presented in Table 5. Based on the available standards, three sugars (fructose, glucose, and sucrose) were quantified in all samples, except for BPr, where sucrose was absent. Fructose was the predominant monosaccharide in the BB and BH samples, while glucose was the main simple sugar in the BP and BPr samples.

### 3.6. Phenolics Profile

Phenolics, recognized as key bioactive compounds in all bee products, were identified and quantified through LC-ESI-MS/MS analysis. The results are presented in Table 6.

A total of 28 phenolics were quantified across the different bee products, with BB containing 27 phenolics, BH containing 26, BP having 25, and BPr containing 23. The total amounts of phenolics in different samples (based on fresh weight) were as follows: 1097.057 mg/kg (BB), 68.823 mg/kg (BH), 5329.995 mg/kg (BP), and 14,858.87 mg/kg (BPr). The obtained results for phenolic quantification are in line with the results for TPC obtained by spectrophotometry. Among the quantified compounds, there were significant differences between the predominant compounds and the amount of the most abundant compounds depending on the bee product analyzed. In the case of BB, the three most predominant compounds were luteolin (169.117 mg/kg), rutin (136.10 mg/kg), and *trans*-caffeic acid (119.204 mg/kg). Regarding BH, the most predominant phenolics were as follows: *trans*-caffeic acid (38.890 mg/kg), pinobanksin (20.111 mg/kg), and chrysin (1.135 mg/kg). BP had the following compounds as predominant: hyperoside, i.e., quercetin-3-*O*-galactoside (1582.495 mg/kg); myricetin (1574.07 mg/kg); and naringenin (793.981 mg/kg). Finally, the phenolic profile of BPr was quite different compared to other bee products, with pinobaksin (11,698.49 mg/kg) as the most predominant phenolic compound, followed by naringenin (1530.43 mg/kg) and chrysin (338.26 mg/kg). In contrast, some of the phenolic compounds were either absent or present only in traces such as artepillin C in the case of BB (0.035 mg/kg), BH (0.055 mg/kg) BP (0.036 mg/kg), and BPr (0.11 mg/kg), as well as epicatechin gallate (0.097 mg/kg) in BB and (0.077 mg/kg) BH, alongside catechin in BPr (0.031 mg/kg) and BH (0.41 mg/kg).

### 3.7. Enzyme Inhibition

Table 7, Table 8, Table 9, Table 10, Table 11 and Table 12 show that inhibition % values of the samples and standards on the enzymes were increased with increasing concentration. Lower (IC_50_) values indicate greater inhibitory activity.

Table 7 reveals that the α-amylase inhibitory activities of the bee products and standard acarbose followed this order: acarbose > BP > BH > BB > BPr. Among the samples, BP displayed the greatest inhibitory activity with the lowest IC_50_ value for α-amylase inhibition at 10.60 ± 0.83 mg/mL. Table 7 also shows α-glucosidase inhibitory activities of the samples and standard. According to this study, the α-glucosidase inhibitory activities of the samples decreased in the following order: acarbose > BPr > BP > BB > BH. In this case, propolis had the lowest IC_50_ value for α-glucosidase inhibitor activity among the tested samples (1.68 ± 0.29 mg/mL). These results indicate that all of the samples function as α-amylase and α-glucosidase inhibitors capable of inhibiting α-amylase and α-glucosidase enzyme activity.

The ability of the samples and standard to inhibit lipase and acetylcholinesterase is presented in Table 8. While BPr and BH exhibited lipase inhibitory activity, BB and BP had no anti-lipase activity, as shown in Table 8. Propolis exhibited the highest lipase inhibitory activity (IC_50_ = 4.67 ± 0.28 mg/mL).

Investigation on acetylcholinesterase (AChE) inhibitory activity of the samples has revealed dose-dependent inhibitory activities, as presented in Table 8. Propolis once again stood out, showing the highest AChE inhibitory activity. The acetylcholinesterase inhibitory activities of the samples decreased in the following order: Tacrin > BPr > BP > BB. BH extract had no acetylcholinesterase inhibitory activity.

The neuraminidase and angiotensin-converting enzyme inhibitions of the samples are represented in Table 9. Neuraminidase inhibition of the samples and standards decreased in the following order: quercetin dihydrate > BPr > BH > BP > BB. All the tested samples exhibited neuraminidase inhibitory activity. Angiotensin-converting enzyme inhibition of the samples and standards decreased in the following order: captopril > BP > BB. Propolis and honey extracts had no angiotensin-converting enzyme inhibitory activity.

The ability of the samples and standard to inhibit urease and trypsin is presented in Table 10. All the tested samples exhibited urease and trypsin inhibitory activities, as shown in Table 10. Propolis had the highest urease inhibitory activity (IC50 = 3.78 ± 0.07 mg/mL). Urease inhibitory activity of the samples and standard decreased in the following order: thiourea > BPr > BB > BP > BH. Investigation of trypsin inhibitory activity demonstrated dose-dependent inhibitory effects, as presented in Table 10. The highest trypsin inhibitory activity was found in the honey extract. The trypsin inhibitory activities of the samples decreased in the following order: BH > tannic acid > BPr > BB > BP.

The tyrosinase and carbonic anhydrase inhibitory activities of the samples were investigated in vitro, with kojic acid and acetazolamide used as reference standards, respectively. In order to quantify the tyrosinase and carbonic anhydrase inhibitory activities, the half maximal inhibitory concentration (IC_50_) values are shown in Table 11. Lower (IC_50_) values indicate greater tyrosinase and carbonic anhydrase inhibitory activities. Bee bread (IC_50_ = 91.61 ± 5.55 mg/mL) and pollen (IC_50_ = 131.17 ± 9.29 mg/mL) exhibited tyrosinase inhibitor activity, while the other samples showed no inhibitory activity compared to the reference standard kojic acid (IC_50_ = 0.084 ± 0.001 mg/mL) in this study.

Propolis (IC_50_ = 0.82 ± 0.04 mg/mL) and pollen (IC_50_ = 1.31 ± 0.06 mg/mL) showed high inhibition against carbonic anhydrase enzyme activity. According to this study, the carbonic anhydrase inhibitory activities of the samples decreased in the following order: acetazolamide > BPr > BP > BB > BH.

All the tested compounds exhibited thioredoxin reductase and adenosine deaminase inhibitory activities (Table 12). The thioredoxin reductase inhibitory activities were ranked as follows: 2,4-dinitrochlorobenzene > BPr > BH > BP > BB.

As shown in Table 12, propolis, honey extract, pollen, and bee bread exhibited adenosine deaminase inhibitory activities. Their IC_50_ values were 3.13 ± 0.23, 39.79 ± 1.31, 57.71 ± 0.76, and 63.58 ± 0.82 mg/mL, respectively, indicating good adenosine deaminase inhibitory activities after EHNA. HCl was used as a positive control (IC_50_ = 0.61 ± 0.05 mg/mL).

## 4. Discussion

The plant source of bee products has a direct effect on their physical, physicochemical, and chemical properties. Therefore, determining the plant sources of bee products is important for standardizing these products [79]. At this point, palynological analysis is the most widely used traditional method to determine the plant source of bee products [24,79,80]. Palynological analyses of honey, pollen, and bee bread samples collected from the same apiary and used in the study were carried out. According to the results obtained, it was determined that the samples showed a similar pollen profile. In general, the plant genera present in the samples predominantly originated from plants belonging to the Asteraceae and Fabaceae families, in accordance with the region’s flora.

Through proximate composition analysis, the distribution of nutrients and the energetic value of examined food ingredients can be determined. In the current study, BP, BH, and BB exhibited the highest protein (BB and BP) and carbohydrate content (BP). Compared to the literature, the BB sample was characterized by significantly higher carbohydrate content (current study: 86.4%, reported range: 33.1–73%) but lower protein content (current study: 5.0%, reported range: 15.3–23.5%) [81]. In the case of BP, the protein content was lower (5.5%) compared to the proposed literature range (10–40%) [82]; however, this parameter is strongly influenced by the botanical origin of the pollen [33]. For the honey sample, carbohydrates (86.8%) and protein content (~0.1%) were comparable to literature values [3], which suggest ~80% for carbohydrates and 0.3% for proteins in blossom honey. Although propolis has been considered a low protein matrix, recent research confirmed noteworthy protein diversity in propolis [83], aligning with significant protein content (13.2%) observed in the BPr sample. However, the BPr (26.5%) and BH (13%) samples stood out in terms of lipid content compared to BP and BB (~4–5%). Propolis is known to be a source of various fatty acids and other lipophilic substances such as terpenes, sterols, and waxy acids [16]. The lipid content in BH was significantly higher in this study compared to available literature (0.2–0.3 g/100 g) [84], though there are limited data on this parameter. When comparing the obtained energetic values (375.3 kcal for BB–520.9 kcal for BPr), it can be concluded that BPr is the richest source of energy. However, the most important factor is energy distribution, which should originate from 10–12% proteins, 30% lipids, and 60% carbohydrates [85].

Food color is one of the most important sensory parameters determining consumer acceptance of a food product. The color parameters of the analyzed samples were determined using the international CIELab (L* a* b*) standard. The L* parameter (luminance or lightness) is expected to range from 0 to 100, while the a* (green to red color) and b* (blue to yellow color) parameters typically range from −120 to 120 [86]. Along with honey, pollen is a bee product whose characteristics are significantly influenced by its color [87]. Pollen color is strongly determined by its botanical origin, resulting in a wide color spectrum, though it is mostly yellowish to orange [87]. The examined BP sample was characterized by an L* value higher than 50, indicating the lightness of the sample, similar to literature reports for other pollen samples [87]. Moreover, the BP sample can be described as exceptionally light due to its H* value being higher than 70 [87]. Similarly, the BH sample also exhibited high lightness, with an H* value close to 70, which is significantly higher compared to several other honey samples (sunflower, rapeseed, polyfloral, mint, thyme, raspberry), where reported H* values ranged from 34.4 to 46.1 [88]. However, contrary to literature data, the BP sample was not characterized by a yellow(ish) color due to its low b* value (28.2), which is significantly lower than the proposed yellowness threshold (b* > 50) [87]. Regarding the C* parameter, which reflects saturation level, all samples, except BP, can be classified as having low saturation, with values close to or lower than 11, as suggested by the literature [87].

General phytochemical composition, determined via several spectrophotometric methods, is one of the most commonly reported parameters for characterizing different bee products. As expected, BPr exhibited the highest TPC value, as it is recognized as an excellent source of various phenolic compounds [16]. Among other products, BP showed significant phenolic content, in accordance with the proposed literature range of 0.5–213 mg/g GAE [82]. However, the BB sample had a significantly lower TPC value than suggested in the literature [81]. A similar trend was observed for the TFC parameter. In contrast to TPC and TFC, the BB sample had a higher carotenoid content than BP, likely due to the fermentation process that occurs in bee bread. Nonetheless, BPr still stood out as the best source of carotenoids in this study. Regarding the BH sample, it exhibited similar TPC but lower TFC values compared to literature data (0.18–0.24 mg/g GAE; 0.17–0.33 mg/g QE) [88] for several types of honey. Differences and/or deviations observed in the samples are likely due to their botanical and geographical origins [25].

Regarding specific sugars present in the samples, BH (in total 72.1 g/100 g) and BP (in total 60.8 g/100 g) stood out. In contrast, a significantly lower amount (in total 26.3 g/100 g) of sugars was observed in BB, likely due to microbial activity during the fermentation process. The most abundant sugar was fructose (BH, BB), i.e., glucose (BP and BPr), across all samples. For BH, the sugar composition was consistent with literature findings [3], with fructose being the most abundant monosaccharide. However, an opposite trend was observed in honey samples from Ethiopia [84], where glucose was more abundant than fructose. In the case of the BP sample, the determined amounts of glucose and fructose were significantly higher than the average content [82] reported for pollen samples (13.4 g/100 g for glucose, 15.4 g/100 g for fructose). However, the opposite trend was noted for sucrose (4.2 g/100 g). Nonetheless, all results for BP fit within the ranges observed in the literature [82], highlighting the importance of both botanical and geographical origin in determining the sugar composition of honey and pollen. The BB sugar composition was in line with literature reports, where fructose was also the predominant monosaccharide [89]. Sucrose was not detected in BPr. In general, sugars are not characteristic compounds in propolis. According to literature, only a few sugars have been identified in propolis, including ribose, fructose, glucitol (sugar alcohol), gulose, talose, sucrose, and glucose [16].

Due to several limitations and interferences, the spectrophotometric determination of phenolic compounds can sometimes yield lower or higher values than expected [90]. Therefore, a detailed phenolic profile for all bee products was determined using the LC-ESI-MS/MS method. In accordance with the TPC results, the highest total individual phenolic content (expressed on fresh weight) was found in the BPr sample (14858.87 mg/kg). Among the 28 monitored phenolic compounds (based on available standards), as many as 10 were present in the BP sample in the highest amounts. Additionally, six phenolic compounds were predominantly quantified in the BB sample, with phenolic acids (*p*-coumaric acid, *trans*-caffeic acid, and *trans*-ferulic acid) dominating. This was likely due to the fermentation process, which may have liberated phenolic acids from plant cell walls or glycosides, similar to what occurs during in vitro digestion [91]. Eleven phenolics were predominant in BPr: CAPE, gallic acid, naringenin, pinocembrin, kaempferol, chlorogenic acid, neochlorogenic acid, catechol, and pinobanksin, with pinobanksin being the most abundant. This flavanonol, which is commonly found in propolis, is known for its antibacterial properties [92]. Similarly, the BH sample was characterized by the predominance of this flavonoid (20.1 mg/kg) and *trans*-caffeic acid (38.9 mg/kg), while all other phenolics were quantified in significantly lower amounts (0.024–2.19 mg/kg). However, the most interesting results in this study are related to the quantification of CAPE* and Artepillin C in all bee products, although previously these compounds were almost exclusively reported as propolis components [16,92].

In many studies, bee bread, propolis, honey, and pollen extracts have exhibited the ability to act as antidiabetic, anti-obesity, and anti-Alzheimer’s agents by inhibiting related enzymes, including α-amylase [93,94], α-glucosidase [93,95], lipase [96], AChE [97], neuraminidase [98], ACE [99], urease [97], trypsin [100,101], tyrosinase [102], CA [103], TrxR [104], and ADA [105]. In this study, the in vitro inhibition effects of α-amylase and α-glucosidase, enzymes associated with diabetes mellitus, were investigated. Both previous studies and the current results indicate a significant relationship between the phenolic content of bee products and their AChE inhibition activity, suggesting that phenolic substances may play a key role in enzyme inhibition [97,106]. The obtained inhibition values were compared to those of 9-amino-1,2,3,4-tetrahydro-acridine hydrochloride (tacrine), a commonly used AChE inhibitor in Alzheimer’s disease treatment [107]. The IC_50_ value of tacrine was 0.000081 mg/mL, smaller than the inhibition results of the examined bee product samples. All bee products showed trypsin inhibitory activities ranging from 0.0035 to 24.16 mg/mL for trypsin. The BH extract showed the highest inhibition (0.0035 mg/mL), even stronger than tannic acid, the positive control (0.029 mg/mL). The TrxR enzyme is an enzyme that inhibits the apoptotic pathway, which in turn causes cancer development. For this reason, inhibition of TrxR enzyme is important in preventing various types of cancer [108]. Numerous anti-cancer medications block TrxR [109], and novel drug candidates are being developed on a regular basis. Synthesized substances must undergo long and difficult tests to be used as medicine. For this reason, the current study focused on bee products as natural remedies, similar to Akkemik and Akbulut’s study, which examined the inhibition effects of honey products on TrxR [104]. When examining the inhibition effects of BH, BB, BP, and BPr extracts on TrxR enzyme activities, the strongest TrxR inhibitory effect was seen in the BPr extract (IC_50_ = 0.58 ± 0.03 mg/mL). Consequently, all the bee products exhibited significant potential for inhibiting enzymes such as α-amylase, α-glucosidase, neuraminidase, urease, trypsin, CA, TrxR, and ADA. It can be assumed that the high level of enzyme inhibition is due to the phenolics present in these bee products. The investigated enzyme inhibition activities of these bee products probably depend on their composition. Indeed, the difference in the chemical composition of different bee products such as honey, pollen, propolis, bee bread, royal jelly, and apilarnil can also be observed in different studies conducted in the literature [10,110,111,112,113,114]. However, further studies are needed to confirm observed regularity and properties.

## 5. Conclusions

One apiary/beehive can be treated as an excellent source of different beneficial products for humans. The current study examined honey, bee pollen, bee bread, and propolis obtained from the same apiary in order to determine their potential as a source of nutrients and different bioactive compounds. It was observed that bee pollen is an excellent source of different nutrients, while propolis confirmed the highest quantity of phenolic compounds. For further examination, twelve different enzymatic assays were implemented on collected bee products. It was observed that propolis exhibited the highest bioactivity in most of them, while honey and bee pollen also can act as anti-inflammatory agents in some of the enzymatic reactions triggered in our body. Further research should include more different locations with different botanical/geographical origin of bee products in order to determine possible regularity.

## Figures and Tables

**Table 1:** Botanical origins of the examined bee product samples (%).

Family	Family/Genus	BH	BP	BB
Asteraceae	Asteraceae	16.2	15.0	17.0
	*Achillea* spp.	3.0	2.0	3.0
	*Centaurea* spp.			1.6
	*Aster* spp.			2.4
Boraginaceae	Boraginaceae	4.0		
Brassicaceae	Brassicaceae	9.8	5.0	
	*Brassica* spp.			
Campanulaceae	*Campanula* spp.	1.0		
Fabaceae	Fabaceae	12.2	24.2	22.0
	*Lotus* spp.	3.8	0.8	3.0
	*Astragalus* spp.	13.6	8.2	10.0
	*Trifolium* spp.	6.4	15	9.0
	*Medicago* spp.	6.2	1.0	3.0
	*Onobrychis* spp.	5.8	9.2	8.0
	*Melilotus* spp.	-	5.8	6.6
Lamiaceae	Lamiaceae	4.0	3.0	7.0
	*Lamium* spp.	2.2		
Malvaceae	Malvaceae	0.8		
Polygonaceae	*Rumex* spp.		0.2	
Ranunculaceae	Ranunculaceae	0.6		
Rosaceae	Rosaceae	6.0	7.6	7.4
Salicaceae	Salicaceae	4.4	3.0	

**Table 2:** Results of the proximate composition analysis (% DW).

	Protein * (% DW)	Carbohydrate (% DW)	Lipid (% DW)	Moisture (%)	Ash (% DW)	Energy (kcal)
BB	5.003 ± 0.483 bC	86.443 ± 0.424 aA	4.96 ± 0.32 cC	8.785 ± 0.119 aB	3.594 ± 0.102 aD	375.283 ± 1.636 d
BH	0.079 ± 0.002 cD	86.815 ± 0.619 aA	13.02 ± 0.62 bB	4.564 ± 0.118 bC	0.086 ± 0.002 dD	446.499 ± 3.369 b
BP	5.479 ± 0.028 bB	87.872 ± 0.275 aA	4.227 ± 0.284 cC	3.849 ± 0.108 cC	2.422 ± 0.001 bD	396.047 ± 1.625 c
BPr	13.234 ± 1.626 aC	58.216 ± 1.706 bA	26.5 ± 2.858 aB	0.84 ± 0.01 dD	2.05 ± 0.07 cD	520.942 ± 14.532 a

* All results are expressed as mean ± standard deviation (n = 3). In each column, different lowercase letters (a–d) indicate significant differences between BB, BH, BPr, and BP samples according to Tukey’s test (*p* < 0.05). In each row, different uppercase letters (A–D) indicate significant differences between physicochemical assays according to Tukey’s test (*p* < 0.05). DW: dry weight.

**Table 3:** Results of phytochemical analysis (mg/g FW).

	BB	BH	BP	BPr
Total phenolic * (mg GAE/g)	4.96 ± 0.54 b	0.43 ± 0.04 b	7.07 ± 0.64 b	85.27 ± 6.06 a
Total flavonoid (mg QE/g)	0.8 ± 0.02 b	0.04 ± 0.01 b	1.09 ± 0.03 b	44.8 ± 1.54 a
Total proanthocyanidin (mg CE/g)	1.22 ± 0.03 b	0.13 ± 0.03 d	0.49 ± 0.06 c	1.52 ± 0.04 a
Total carotenoid (μg/g)	69.47 ± 0.36 b	2.69 ± 0.08 d	43.52 ± 0.13 c	208.69 ± 6.66 a
Total anthocyanin (μg/g)	nd	nd	nd	nd
CUPRAC (mg TE/g)	6.2 ± 0.24 b	0.58 ± 0.03 c	6.81 ± 0.39 b	133.84 ± 2.59 a
DPPH^•^ (mg TE/g)	9.83 ± 1.01 b	1.26 ± 0.03 c	11.88 ± 1.01 b	71.34 ± 0.91 a
ABTS^•+^ (mg TE/g)	4.56 ± 0.37 b	3.33 ± 0.42 b	5.13 ± 0.63 b	71.29 ± 2.47 a

* All results are expressed as mean ± standard deviation (n = 3). nd stands for not detected. In each row, different lowercase letters (a–d) indicate significant differences between BB, BH, BPr, and BP samples according to Tukey’s test (*p* < 0.05). GAE: gallic acid equivalent, QE: quercetin equivalent, CE: catechin equivalent, TE: Trolox equivalent, FW: fresh weight.

**Table 4:** Instrumental color data (CIE L*a*b*).

Color						
	L*	a*	b*	C*	H*	∆E
BB	43.12 ± 0.78 b	7.34 ± 0.8 b	8.39 ± 1.26 b	11.15 ± 1.47 b	48.68 ± 1.25 d	54.49 ± 0.77 b
BH	38.7 ± 0.62 c	3.54 ± 0.1 c	8.68 ± 0.59 b	9.38 ± 0.58 bc	67.76 ± 0.79 b	58.52 ± 0.5 a
BP	59.29 ± 0.56 a	8.74 ± 0.31 a	28.25 ± 0.59 a	29.57 ± 0.6 a	72.81 ± 0.57 a	48.4 ± 0.04 c
BPr	41.8 ± 1.02 b	3.24 ± 0.06 c	5.84 ± 0.2 c	6.68 ± 0.18 c	60.92 ± 0.69 c	54.89 ± 0.98 b

L*: lightness; a*: green-red; b*: blue-yellow; C*: chroma; H*: hue angle; ∆E: the difference between two colors in the Lab* space. In each column, different lowercase letters (a–d) indicate significant differences between BB, BH, BPr, and BP samples according to Tukey’s test (*p* < 0.05).

**Table 5:** Sugar content of bee bread, honey, pollen, and propolis samples by UHPLC-RID (g/100 g FW).

Compound	BB	BH	BP	BPr
Fructose *	14.697 ± 1.171 aC	36.66 ± 2.921 aA	22.941 ± 1.828 bB	2.663 ± 0.212 bD
Glucose	10.436 ± 0.574 bB	33.907 ± 1.866 aA	36.602 ± 2.014 aA	7.664 ± 0.422 aB
Sucrose	1.165 ± 0.056 cB	1.846 ± 0.089 bA	1.261 ± 0.061 cB	nd

* All results are expressed as mean ± standard deviation (n = 3). nd stands for not detected; FW—fresh weight. In each column, different lowercase letters (a–c) indicate significant differences between fructose, glucose, and sucrose according to Tukey’s test (*p* < 0.05). In each row, different uppercase letters (A–D) indicate significant differences between BB, BH, BPr, and BP samples according to Tukey’s test (*p* < 0.05).

**Table 6:** Quantification of phenolics through LC-ESI-MS/MS (mg/kg FW).

Phenolic Groups	Compound	BB	BH	BP	BPr
Flavan 3-ols	(-)-Epicatechin **	0.472 ± 0.014 cM	0.998 ± 0.085 bDEF	2.829 ± 0.297 aJK	2.19 ± 0.22 cM
	Catechin	nd	0.41 ± 0.043 aDEFG	nd	0.31 ± 0.04 bM
	Epicatechin gallate	0.097 ± 0.005 bM	0.077 ± 0.003 bFG	0.395 ± 0.011 aJK	nd
Flavones	Apigenin	18.794 ± 0.091 bIJKL	0.247 ± 0.026 cDEFG	134.565 ± 0.893 aD	3.81 ± 0.33 cM
	Chrysin	25.91 ± 1.105 cHIJ	1.135 ± 0.074 dD	97.415 ± 0.689 aDE	338.26 ± 2.51 bC
	Galangin	31.655 ± 0.155 bGHI	0.136 ± 0.01 cEFG	89.256 ± 4.114 aE	6.87 ± 0.6 cM
	Hyperoside	41.999 ± 1.368 bEFG	0.024 ± 0.001 cG	1582.495 ± 8.047 aA	32.2 ± 1.24 cL
	Luteolin	169.117 ± 4.508 bA	0.03 ± 0.005 cG	240.86 ± 6.073 aC	7.29 ± 0.13 cM
Flavanones	Hesperidin	47.049 ± 0.132 aEF	0.178 ± 0.004 cEFG	34.311 ± 2.36 bHIJK	7.77 ± 0.53 cM
	Naringenin	39.195 ± 0.19 cEFGH	0.364 ± 0.012 dDEFG	793.981 ± 8.138 aB	1530.43 ± 7.08 bB
	Pinocembrin	19.417 ± 0.523 cIJKL	0.569 ± 0.05 dDEFG	94.274 ± 1.007 aDE	297.42 ± 1.07 bD
Flavonols	Kaempferol	45.065 ± 3.066 bEFG	0.096 ± 0.005 dFG	69.057 ± 2.774 aEFGH	104.89 ± 2.48 cH
	Myricetin	87.487 ± 1.213 bD	0.139 ± 0.03 cEFG	1574.07 ± 50.401 aA	62.81 ± 3.6 cIJ
	Quercetin	6.663 ± 0.189 bLM	0.326 ± 0.017 cDEFG	17.034 ± 0.679 aIJK	3.91 ± 0.52 cM
	Quercitrin	36.774 ± 0.822 bFGH	0.097 ± 0.007 cFG	237.123 ± 3.389 aC	44.84 ± 1.66 cKL
	Rutin	136.1 ± 2.991 aB	0.266 ± 0.039 dDEFG	82.347 ± 1.363 bEF	78.21 ± 3.66 cI
Phenolic acids	Artepillin C	0.035 ± 0.001 bM	0.055 ± 0.001 aFG	0.036 ± 0.002 bK	0.11 ± 0.01 cM
	CAPE *	6.379 ± 0.164 cLM	0.034 ± 0.001 dG	79.333 ± 0.988 aEFG	196.41 ± 1.74 bE
	Chlorogenic acid	22.188 ± 0.125 aIJK	1.086 ± 0.007 cDE	nd	175.93 ± 2.55 bF
	Gallic acid	0.865 ± 0.043 cM	0.252 ± 0.02 dDEFG	1.187 ± 0.04 bJK	50.73 ± 0.32 aJK
	Neochlorogenic acid	0.753 ± 0.031 bM	0.205 ± 0.01 cDEFG	0.236 ± 0.042 cJK	72.91 ± 0.2 aI
	*p*-Coumaric acid	82.467 ± 4.773 aD	0.572 ± 0.033 cDEFG	40.776 ± 1.746 bGHIJ	nd
	Protocatechuic acid	78.347 ± 1.788 aD	2.191 ± 0.044 cC	27.915 ± 2.081 bIJK	2.97 ± 0.49 cM
	*trans*-Cinnamic acid	7.407 ± 0.204 bLM	nd	35.004 ± 0.331 aHIJK	nd
	*trans*-Caffeic acid	119.204 ± 15.999 aC	38.89 ± 1.22 bA	46.556 ± 0.915 bFGHI	nd
	*trans*-Ferulic acid	13.419 ± 0.286 aJKLM	0.335 ± 0.041 cDEFG	10.194 ± 0.534 bIJK	nd
Benzenediols	Catechol	9.306 ± 0.595 bKLM	nd	nd	140.11 ± 0.668 aG
Dihyroflavonols	Pinobanksin	50.893 ± 1.17 bE	20.111 ± 0.211 dB	38.746 ± 1.028 cGHIJK	11,698.49 ± 1.734 aA
Total		**1097.057**	**68.823**	**5329.995**	**14,858.87**

* CAPE: caffeic acid phenethyl ester; nd stands for not detected; FW: fresh weight. ** All results are expressed as mean ± standard deviation (n = 3). In each column, different uppercase letters (A–M) indicate significant differences between phenolics content according to Tukey’s test (*p* < 0.05). In each row, different lowercase letters (a–d) indicate significant differences between BB, BH, BPr, and BP samples according to Tukey’s test (*p* < 0.05).

**Table 7:** Inhibitory activities of samples on α-amylase and α-glucosidase.

Samples/ Standard	α-Amylase			α-Glucosidase		
	Concentration (mg/mL)	Inhibition (%) *	IC_50_ (mg/mL) *	Concentration (mg/mL)	Inhibition (%) *	IC_50_ (mg/mL) *
BB	30	54.90 ± 2.99	25.36 ± 1.84	60	81.30 ± 3.77	33.06 ± 2.97
	15	37.47 ± 1.96		50	67.81 ± 6.21	
	7.5	27.47 ± 1.53		40	62.35 ± 5.98	
	5	20.93 ± 0.70		30	43.08 ± 3.14	
	0.5	10.78 ± 1.54		0.5	13.64 ± 0.29	
BH	12.5	47.01 ± 6.79	16.02 ± 2.22	75	46.27 ±4.96	91.08 ± 12.73
	10	29.49 ± 1.26		50	24.92 ±1.50	
	5	19.66 ± 1.83		5	19.62 ± 2.22	
	0.5	13.28 ± 1.44		0.5	9.44 ± 1.18	
	0.05	10.51 ± 2.10		0.05	4.62 ± 1.15	
BP	12.5	61.77 ± 1.02	10.60 ± 0.83	25	52.25 ± 1.73	23.28 ± 1.03
	10	38.99 ± 5.33		20	43.90 ± 2.93	
	2.5	32.46 ± 2.18		15	34.23 ± 4.23	
	0.1	24.63 ± 1.56		10	23.49 ± 7.18	
	0.01	16.37 ± 2.19		7.5	16.54 ± 5.57	
BPr	50	63.80 ± 3.33	40.52 ± 1.29	2.5	63.22 ± 5.03	1.68 ± 0.29
	40	45.56 ± 1.74		0.5	43.30 ± 13.81	
	30	37.08 ± 0.54		0.25	23.63 ± 4.72	
	15	24.05 ± 0.78		0.05	12.47 ± 1.44	
	10	12.50 ± 2.69		0.025	6.86 ± 1.62	
Acarbose **	0.06	54.81 ± 2.86	0.052 ± 0.003	1	83.91 ± 0.68	0.63 ± 0.01
	0.045	43.86 ± 1.83		0.5	24.47 ± 0.82	
	0.03	36.03 ± 1.83		0.1	17.34 ± 2.45	
	0.015	25.44 ± 1.42		0.01	8.87 ± 0.27	
	0.005	6.04 ± 1.46		0.001	2.35 ± 0.76	

* Mean ± SD of triplicate values; ** acarbose is used as a positive control. Abbreviations used: BB—bee bread, BH—bee honey, BP—bee pollen, BPr—bee propolis.

**Table 8:** Inhibitory activities of samples on lipase and acetylcholinesterase.

Samples/ Standard	Lipase			Acetylcholinesterase		
	Concentration (mg/mL)	Inhibition (%) *	IC_50_ (mg/mL) *	Concentration (mg/mL)	Inhibition (%) *	IC_50_ (mg/mL) *
BB	60	nd	-	50	44.02 ± 1.33	64.43 ± 5.06
	30			40	30.83 ± 4.61	
	7.5			30	25.68 ± 1.45	
	5			5	17.64 ± 1.93	
	0.5			1	4.19 ± 0.81	
BH	25	34.47 ± 1.20	42.21 ± 0.79	100	nd	-
	12.5	19.08 ± 0.87		75		
	5	16.61 ± 0.17		5		
	0.5	11.27 ± 0.41		0.1		
	0.05	8.46 ± 2.19		0.005		
BP	60	nd	-	20	59.94 ± 1.21	14.82 ± 0.38
	30			10	41.20 ± 0.47	
	7.5			5	30.43 ± 0.72	
	1			1	19.37 ± 0.25	
	0.1			0.1	5.57 ± 0.82	
BPr	7.5	62.21 ± 0.21	4.67 ± 0.28	10	51.90 ± 4.86	9.29 ± 0.90
	0.5	48.82 ± 0.83		5	34.15 ± 0.36	
	0.25	33.24 ± 5.30		2.5	25.55 ± 1.59	
	0.05	23.63 ± 0.45		1	18.34 ± 0.14	
	0.0005	13.97 ± 1.77		0.5	9.74 ± 0.75	
Orlistat **	0.005	72.60 ± 0.32	0.00245 ± 0.00010			
	0.0025	56.19 ± 2.50				
	0.001	38.43 ± 1.79				
	0.0005	32.20 ± 0.31				
	0.0001	18.43 ± 0.61				
Tacrin **				0.0001	54.36 ± 0.04	0.000081 ± 0.000002
				0.00001	45.33 ± 4.81	
				0.000001	26.56 ± 3.01	
				0.0000001	16.98 ± 1.34	
				0.00000001	8.28 ± 0.58	

* Mean ± SD of triplicate values; ** compounds used as positive controls; nd means not detected. Abbreviations used: BB—bee bread, BH—bee honey, BP—bee pollen, BPr—bee propolis.

**Table 9:** Inhibitory activities of samples on neuraminidase and angiotensin-converting enzyme.

Samples/ Standard	Neuraminidase			Angiotensin Converting Enzyme		
	Concentration (mg/mL)	Inhibition (%) *	IC_50_ (mg/mL) *	Concentration (mg/mL)	Inhibition (%) *	IC_50_ (mg/mL) *
BB	2	60.50 ± 0.24	1.28 ± 0.05	70	60.45 ± 1.64	56.05 ± 1.05
	1	49.35 ± 1.23		60	50.45 ± 1.64	
	0.5	43.24 ± 2.25		40	40.47 ± 0.81	
	0.1	25.51 ± 1.21		30	33.33 ± 2.84	
	0.01	7.63 ± 5.21		10	7.29 ± 0.71	
BH	0.1	56.99 ± 2.79	0.08 ± 0.01	100	nd	-
	0.01	46.80 ± 0.84		1		
	0.004	22.96 ±0.86		0.01		
	0.002	17.88 ± 4.18		0.001		
	0.0001	9.86 ± 0.01		0.0001		
BP	2	67.18 ± 0.76	1.19 ± 0.05	50	47.44 ± 1.23	55.71 ± 1.64
	1	46.91 ± 1.42		40	33.80 ± 1.88	
	0.5	36.70 ± 0.95		30	26.20 ± 0.27	
	0.01	27.09 ± 0.93		10	8.48 ± 0.95	
	0.001	20.53 ± 2.81		1	2.88 ± 0.69	
BPr	0.05	73.03 ± 4.09	0.03 ± 0.01	5	nd	-
	0.0375	57.20 ± 0.64		1		
	0.025	30.77 ± 2.42		0.1		
	0.005	22.70 ± 0.53		0.001		
	0.0001	13.51 ± 1.30		0.0001		
Quercetin dihydrate **	0.0005	57.12 ± 5.40	0.00035 ± 0.00005			
	0.0001	49.00 ± 1.10				
	0.00005	34.41 ± 1.72				
	0.00001	23.84 ± 1.63				
	0.000001	17.00 ± 3.57				
Captopril **				0.000025	66.96 ± 3.34	0.0000092 ± 0.0000001
				0.00001	54.37 ± 0.51	
				0.0000075	40.44 ± 1.94	
				0.000005	32.44 ± 0.44	
				0.000001	13.68 ± 2.20	

* Mean ± SD of triplicate values, ** compounds used as positive controls; nd means not detected. Abbreviations used: BB—bee bread, BH—bee honey, BP—bee pollen, BPr—bee propolis.

**Table 10:** Inhibitory activities of samples on urease and trypsin.

Samples/ Standard	Urease			Trypsin		
	Concentration (mg/mL)	Inhibition (%) *	IC_50_ (mg/mL) *	Concentration (mg/mL)	Inhibition (%) *	IC_50_ (mg/mL) *
BB	30	58.23 ± 2.28	27.83 ± 1.05	10	43.84 ± 1.97	10.63 ± 0.32
	15	46.16 ± 1.53		5	36.08 ± 1.49	
	7.5	31.01 ± 0.82		2.5	16.79 ± 2.52	
	5	23.92 ± 3.04		1	11.75 ± 1.59	
	0.5	14.96 ± 3.82		0.1	5.63 ± 1.58	
BH	50	57.97 ± 5.61	40.89 ± 4.79	0.001	20.07 ± 3.70	0.0035 ± 0.0009
	25	36.54 ± 0.24		0.0001	18.40 ± 2.22	
	0.5	24.94 ± 0.93		0.00001	13.99 ± 1.86	
	0.05	18.17 ± 0.63		0.000001	7.31 ± 1.06	
	0.005	9.03 ± 2.19		0.0000001	3.95 ± 1.08	
BP	30	46.92 ± 1.31	30.17 ± 1.14	30	57.69 ± 1.03	24.16 ± 1.20
	25	35.38 ± 1.50		20	42.38 ± 3.10	
	20	27.08 ± 0.73		10	34.48 ± 3.84	
	10	19.19 ± 0.67		5	14.53 ± 2.81	
	1	8.80 ± 1.92		1	3.06 ± 0.88	
BPr	5	57.03 ± 0.12	3.78 ± 0.07	1	54.28 ± 3.36	0.80 ± 0.05
	2.5	43.38 ± 0.75		0.5	44.53 ± 0.46	
	0.5	35.49 ± 4.38		0.1	29.25 ± 0.78	
	0.025	12.75 ± 0.49		0.05	19.20 ± 3.55	
	0.005	6.38 ± 2.45		0.01	12.59 ±.14	
Thiourea **	0.25	67.62 ± 0.85	0.16 ± 0.01			
	0.1	40.69 ± 1.47				
	0.05	23.21 ± 0.09				
	0.01	18.52 ± 0.55				
	0.001	4.79 ± 0.54				
Tannic acid **				0.05	78.65 ± 1.55	0.029 ± 0.001
				0.025	47.76 ± 0.74	
				0.01	23.43 ± 0.99	
				0.005	15.27 ± 0.81	
				0.001	11.67 ± 0.11	

* Mean ± SD of triplicate values; ** compounds used as positive controls. Abbreviations used: BB—bee bread, BH—bee honey, BP—bee pollen, BPr—bee propolis.

**Table 11:** Inhibitory activities of samples on tyrosinase and carbonic anhydrase.

Samples/ Standard	Tyrosinase			Carbonic Anhydrase		
	Concentration (mg/mL)	Inhibition (%) *	IC_50_ (mg/mL) *	Concentration (mg/mL)	Inhibition (%) *	IC_50_ (mg/mL) *
BB	100	54.93 ± 4.16	91.61 ± 5.55	30	54.61 ± 0.71	22.13 ± 0.58
	75	40.65 ± 3.40		15	49.53 ± 0.92	
	50	27.38 ± 0.29		7.5	37.97 ± 1.26	
	25	20.75 ± 2.12		5	23.74 ± 2.66	
	10	5.44 ± 2.62		0.5	9.54 ± 2.08	
BH	100	N.D.	-	75	52.66 ± 1.78	65.72 ± 2.32
	10			50	42.16 ± 0.33	
	1			25	36.26 ± 0.65	
	0.1			12.5	27.53 ± 2.52	
	0.01			5	14.04 ± 0.69	
BP	75	30.67 ± 2.15	131.17 ± 9.29	1	45.66 ± 2.87	1.31 ± 0.06
	50	21.99 ± 0.81		0.75	29.34 ± 3.91	
	25	15.60 ± 1.11		0.5	21.70 ± 2.67	
	10	10.46 ± 2.15		0.1	19.62 ± 3.91	
	1	3.55 ± 1.34		0.01	10.42 ± 0.90	
BPr	100	nd	-	1	63.74 ± 3.16	0.82 ± 0.04
	10			0.75	40.51 ± 0.96	
	1			0.5	33.81 ± 0.19	
	0.1			0.05	23.44 ± 2.36	
	0.001			0.01	13.92 ± 1.25	
Kojic acid **	0.5	99.58 ± 0.01	0.084 ± 0.001			
	0.1	59.58 ± 0.42				
	0.05	35.63 ± 1.46				
	0.01	10.63 ± 1.46				
	0.001	2.29 ± 0.21				
Acetazolamide **				0.0005	76.67 ± 1.19	0.000291 ± 0.000004
				0.00025	49.27 ± 0.32	
				0.0001	33.24 ± 0.97	
				0.000075	16.52 ± 0.92	
				0.00005	4.76 ± 0.57	

* Mean ± SD of triplicate values; ** compounds used as positive controls L; nd means not detected. Abbreviations used: BB—bee bread, BH—bee honey, BP—bee pollen, BPr—bee propolis.

**Table 12:** Inhibitory activities of samples on thioredoxin reductase and adenosine deaminase.

Samples/ Standard	Thioredoxin Reductase			Adenosine Deaminase		
	Concentration (mg/mL)	Inhibition (%) *	IC_50_ (mg/mL) *	Concentration (mg/mL)	Inhibition (%) *	IC_50_ (mg/mL) *
BB	0.5	23.34 ± 1.25	1.21 ± 0.08	75	56.05 ± 0.33	63.58 ± 0.82
	0.25	18.99 ± 2.23		50	41.31 ± 0.41	
	0.125	15.29 ± 1.19		25	32.09 ± 3.29	
	0.05	8.00 ± 3.32		10	23.60 ± 3.39	
	0.005	5.43 ± 1.72		5	14.60 ± 0.85	
BH	0.5	26.00 ± 1.97	1.07 ± 0.19	30	35.60 ± 1.54	39.79 ± 1.31
	0.25	14.99 ± 0.83		25	27.01 ± 1.43	
	0.125	9.20 ± 2.08		20	22.19 ± 1.40	
	0.05	3.66 ± 1.59		15	15.14 ± 1.04	
	0.005	1.81 ± 0.69		10	4.81 ± 0.89	
BP	0.5	27.07 ± 2.87	1.11 ± 0.08	100	86.70 ± 1.59	57.71 ± 0.76
	0.25	23.80 ± 0.30		75	54.19 ± 2.30	
	0.125	18.00 ± 1.40		50	40.38 ± 4.53	
	0.05	12.87 ± 2.91		25	31.40 ± 0.99	
	0.005	9.48 ± 2.10		5	13.72 ± 2.30	
BPr	0.25	25.30 ± 0.52	0.58 ± 0.03	5	80.13 ± 3.63	3.13 ± 0.23
	0.125	21.50 ± 0.83		4	72.79 ± 1.64	
	0.05	16.09 ± 0.00		3	48.78 ± 5.34	
	0.005	10.80 ± 0.46		2	22.56 ± 4.93	
	0.0005	6.00 ± 0.62		1	13.39 ± 6.44	
2,4-Dinitrochlorobenzene (CDNB) **	0.01	23.47 ± 1.47	0.028 ± 0.002			
	0.005	18.83 ± 0.27				
	0.0001	14.33 ± 0.04				
	0.00005	9.00 ± 0.69				
	0.00001	5.48 ± 0.41				
EHNA.HCl **				0.3	31.15 ± 0.01	0.61 ± 0.05
				0.03	25.58 ± 0.82	
				0.003	19.23 ± 3.81	
				0.0003	14.04 ± 4.08	
				0.00003	1.92 ± 1.09	

* Mean ± SD of triplicate values; ** compounds used as positive controls. Abbreviations used: BB—bee bread, BH—bee honey, BP—bee pollen, BPr—bee propolis.

## Data Availability

The data that support the findings of the current study are available from the corresponding author Y.C. Gercek upon reasonable request.

## Supplementary Materials

The following supporting information can be downloaded at: https://www.mdpi.com/article/10.3390/antiox13121483/s1, Table S1: The quantitation and confirm ions of phenolic compound standards.

## References

[1] Maicelo-Quintana J.L., Reyna-Gonzales K., Balcázar-Zumaeta C.R., Auquiñivin-Silva E.A., Castro-Alayo E.M., Medina-Mendoza M., Cayo-Colca I.S., Maldonado-Ramirez I., Silva-Zuta M.Z. (2024). Potential Application of Bee Products in Food Industry: An Exploratory Review. Heliyon.

[2] Crane E. (1983). The Archaeology of Beekeeping.

[3] Bogdanov S., Jurendic T., Sieber R., Gallmann P. (2008). Honey for Nutrition and Health: A Review. J. Am. Coll. Nutr..

[4] Calderone N.W., Johnson B.R. (2002). The Within-nest Behaviour of Honeybee Pollen Foragers in Colonies with a High or Low Need for Pollen. Anim. Behav..

[5] Almeida-Muradian L.B., Pamplona L.C., Coimbra S., Barth O.M. (2005). Chemical Composition and Botanical Evaluation of Dried Bee Pollen Pellets. J. Food Compos. Anal..

[6] Human H., Nicolson S.W. (2006). Nutritional Content of Fresh, Bee-collected and Stored Pollen of *Aloe greatheadii* var. davyana (Asphodelaceae). Phytochemistry.

[7] Ares A.M., Valverde S., Bernal J.L., Nozal M.J., Bernal J. (2018). Extraction and Determination of Bioactive Compounds from Bee Pollen. J. Pharm. Biomed. Anal..

[8] Barbieri D., Gabriele M., Summa M., Colosimo R., Leonardi D., Domenici V., Pucci L. (2020). Antioxidant, Nutraceutical Properties, and Fluorescence Spectral Profiles of Bee Pollen Samples from Different Botanical Origins. Antioxidants.

[9] Morris A.L., Mohiuddin S.S. (2024). Biochemistry, Nutrients.

[10] Mărgăoan R., Stranț M., Varadi A., Topal E., Yücel B., Cornea-Cipcigan M., Campos M.G., Vodnar D.C. (2019). Bee Collected Pollen and Bee Bread: Bioactive Constituents and Health Benefits. Antioxidants.

[11] Nagai T., Nagashima T., Myoda T., Inoue R. (2004). Preparation and Functional Properties of Extracts from Bee Bread. Food/Nahrung.

[12] Golubkina N.A., Sheshnitsan S.S., Kapitalchuk M.V., Erdenotsogt E. (2016). Variations of Chemical Element Composition of Bee and Beekeeping Products in Different Taxons of the Biosphere. Ecol. Indic..

[13] Krell R. (1996). Value-Added Products from Beekeeping.

[14] Vásquez A., Olofsson T.C. (2009). The Lactic Acid Bacteria Involved in the Production of Bee Pollen and Bee Bread. J. Apic. Res..

[15] Atkin S.L., Barrier S., Cui Z., Fletcher P.D., Mackenzie G., Panel V., Sol V., Zhang X. (2011). UV and Visible Light Screening by Individual Sporopollenin Exines Derived from *Lycopodium clavatum* (Club moss) and *Ambrosia trifida* (Giant ragweed). J. Photochem. Photobiol. B Biol..

[16] Anjum S.I., Ullah A., Khan K.A., Attaullah M., Khan H., Ali H., Bashir M.A., Tahir M., Ansari M.J., Ghramh H.A. (2019). Composition and Functional Properties of Propolis (Bee glue): A Review. Saudi J. Biol. Sci..

[17] Nainu F., Masyita A., Bahar M.A., Raihan M., Prova S.R., Mitra S., Emran T.B., Simal-Gandara J. (2021). Pharmaceutical Prospects of Bee Products: Special Focus on Anticancer, Antibacterial, Antiviral, and Antiparasitic Properties. Antibiotics.

[18] Clarkson P.M., Thompson H.S. (2000). Antioxidants: What Role Do They Play in Physical Activity and Health?. Am. J. Clin. Nutr..

[19] Meda A., Lamien C.E., Romito M., Millogo J., Nacoulma O.G. (2005). Determination of the Total Phenolic, Flavonoid and Proline Contents in Burkina Fasan Honey, as well as Their Radical Scavenging Activity. Food Chem..

[20] Martinello M., Mutinelli F. (2021). Antioxidant Activity in Bee Products: A Review. Antioxidants.

[21] Al-Kafaween M.A., Alwahsh M., Mohd Hilmi A.B., Abulebdah D.H. (2023). Physicochemical Characteristics and Bioactive Compounds of Different Types of Honey and Their Biological and Therapeutic Properties: A Comprehensive Review. Antibiotics.

[22] Pascoal A., Rodrigues S., Teixeira A., Feás X., Estevinho L.M. (2014). Biological Activities of Commercial Bee Pollens: Antimicrobial, Antimutagenic, Antioxidant and Anti-inflammatory. Food Chem. Toxicol..

[23] Denisow B., Denisow-Pietrzyk M. (2016). Biological and Therapeutic Properties of Bee Pollen: A Review. J. Sci. Food Agric..

[24] Mayda N., Özkök A., Ecem Bayram N., Gerçek Y.C., Sorkun K. (2020). Bee Bread and Bee Pollen of Different Plant Sources: Determination of Phenolic Content, Antioxidant Activity, Fatty Acid and Element Profiles. J. Food Meas. Charact..

[25] Tomás A., Falcão S.I., Russo-Almeida P., Vilas-Boas M. (2017). Potentialities of Beebread as a Food Supplement and Source of Nutraceuticals: Botanical Origin, Nutritional Composition and Antioxidant Activity. J. Apic. Res..

[26] Abouda Z., Zerdani I., Kalalou I., Faid M., Ahami M. (2011). The Antibacterial Activity of Moroccan Bee Bread and Bee-pollen (Fresh and Dried) Against Pathogenic Bacteria. Res. J. Microbiol..

[27] Borycka K., Grabek-Lejko D., Kasprzyk I. (2015). Antioxidant and Antibacterial Properties of Commercial Bee Pollen Products. J. Apic. Res..

[28] Premratanachai P., Chanchao C. (2014). Review of the Anticancer Activities of Bee Products. Asian Pac. J. Trop. Biomed..

[29] Markiewicz-Żukowska R., Naliwajko S.K., Bartosiuk E., Moskwa J., Isidorov V., Soroczyńska J., Borawska M.H. (2013). Chemical Composition and Antioxidant Activity of Beebread, and Its Influence on the Glioblastoma Cell Line (U87MG). J. Apic. Sci..

[30] Sobral F., Calhelha R.C., Barros L., Dueñas M., Tomás A., Santos-Buelga C., Vilas-Boas M., Ferreira I.C. (2017). Flavonoid Composition and Antitumor Activity of Bee Bread Collected in Northeast Portugal. Molecules.

[31] Etebarian A., Alhouei B., Mohammadi-Nasrabadi F., Esfarjani F. (2024). Propolis as a Functional Food and Promising Agent for Oral Health and Microbiota Balance: A Review Study. Food Sci. Nutr..

[32] Kurek-Górecka A., Rzepecka-Stojko A., Górecki M., Otręba M. (2023). Special Issue on Propolis and Other Bee Products: Beneficial Effects on Health and Processing Technology. Appl. Sci..

[33] Campos M.G.R., Bogdanov S., de Almeida-Muradian L.B., Szczesna T., Mancebo Y., Frigerio C., Ferreira F. (2008). Pollen Composition and Standardisation of Analytical Methods. J. Apic. Res..

[34] Nordin A., Sainik N.Q.A.V., Chowdhury S.R., Saim A.B., Idrus R.B.H. (2018). Physicochemical Properties of Stingless Bee Honey from Around the Globe: A Comprehensive Review. J. Food Compos. Anal..

[35] Urcan A., Marghitas L., Dezmirean D., Bobis O., Bonta V., Mureşan C., Mărgăoan R. (2017). Chemical Composition and Biological Activities of Beebread—Review. Bull. Univ. Agric. Sci. Vet. Med. Cluj-Napoca Anim. Sci. Biotechnol..

[36] Wachkoo A.A., Nayik G.A., Uddin J., Ansari M.J. (2024). Honey Bees, Beekeeping and Bee Products.

[37] WHO (2007). Protein and Amino Acid Requirements in Human Nutrition.

[38] Zhang W., Xiao D., Mao Q., Xia H. (2023). Role of Neuroinflammation in Neurodegeneration Development. Signal Transduct. Target. Ther..

[39] Złotek U., Rybczyńska-Tkaczyk K., Michalak-Majewska M., Sikora M., Jakubczyk A. (2020). Potential Acetylcholinesterase, Lipase, α-Glucosidase, and α-Amylase Inhibitory Activity, as well as Antimicrobial Activities, of Essential Oil from Lettuce Leaf Basil (*Ocimum basilicum* L.) Elicited with Jasmonic Acid. Appl. Sci..

[40] Patra J., Singh D., Jain S., Mahindroo N. (2021). Application of Docking for Lead Optimization. Molecular Docking for Computer-Aided Drug Design.

[41] Chen L., Hao G. (2020). The Role of Angiotensin-converting Enzyme 2 in Coronaviruses/influenza Viruses and Cardiovascular Disease. Cardiovasc. Res..

[42] Dolinska M.B., Kovaleva E., Backlund P., Wingfield P.T., Brooks B.P., Sergeev Y.V. (2014). Albinism-Causing Mutations in Recombinant Human Tyrosinase Alter Intrinsic Enzymatic Activity. PLoS ONE.

[43] Sana T., Khan M., Jabeen A., Shams S., Hadda T.B., Begum S., Siddiqui B.S. (2023). Urease and Carbonic Anhydrase Inhibitory Effect of Xanthones from *Aspergillus nidulans*, an Endophytic Fungus of *Nyctanthes arbortristis*. Planta Med..

[44] Zhan X., Wan J., Zhang G., Song L., Gui F., Zhang Y., Li Y., Guo J., Dawra R.K., Saluja A.K. (2019). Elevated Intracellular Trypsin Exacerbates Acute Pancreatitis and Chronic Pancreatitis in Mice. Am. J. Physiol. Gastrointest. Liver Physiol..

[45] Gao Z.-w., Wang X., Zhang H.-z., Lin F., Liu C., Dong K. (2021). The Roles of Adenosine Deaminase in Autoimmune Diseases. Autoimmun. Rev..

[46] Jastrząb A., Skrzydlewska E. (2021). Thioredoxin-dependent system. Application of Inhibitors. J. Enzym. Inhib. Med. Chem..

[47] Sorkun K. (2010). Türkiye’nin Nektarlı Bitkileri, Polenleri ve Balları.

[48] Louveaux J., Maurizio A., Vorwohl G. (1978). Methods of Melissopalynology. Bee World.

[49] Bradford M.M. (1976). A Rapid and Sensitive Method for the Quantitation of Microgram Quantities of Protein Utilizing the Principle of Protein-dye Binding. Anal. Biochem..

[50] Zor T., Selinger Z. (1996). Linearization of the Bradford Protein Assay Increases Its Sensitivity: Theoretical and Experimental Studies. Anal. Biochem..

[51] Mæhre H.K., Dalheim L., Edvinsen G.K., Elvevoll E.O., Jensen I.J. (2018). Protein Determination-Method Matters. Foods.

[52] Bligh E.G., Dyer W.J. (1959). A Rapid Method of Total Lipid Extraction and Purification. Can. J. Biochem. Physiol..

[53] AOAC (1997). Association of Official Analytical Chemists International Official Methods of Analysis.

[54] Yang K., Wu D., Ye X., Liu D., Chen J., Sun P. (2013). Characterization of Chemical Composition of Bee Pollen in China. J. Agric. Food. Chem..

[55] Merrill A.L., Watt B.K. (1973). Energy Value of Foods: Basis and Derivation, Agricultural Handbook.

[56] Magalhães L.M., Santos F., Segundo M.A., Reis S., Lima J.L. (2010). Rapid Microplate High-throughput Methodology for Assessment of Folin-Ciocalteu Reducing Capacity. Talanta.

[57] Chang C.-C., Yang M.-H., Wen H.-M., Chern J.-C. (2002). Estimation of Total Flavonoid Content in Propolis by Two Complementary Colorimetric Methods. J. Food Drug Anal..

[58] Kostić A., Milinčić D.D., Nedić N., Gašić U.M., Špirović Trifunović B., Vojt D., Tešić Ž.L., Pešić M.B. (2021). Phytochemical Profile and Antioxidant Properties of Bee-Collected Artichoke (*Cynara scolymus*) Pollen. Antioxidants.

[59] Lee J., Durst R.W., Wrolstad R.E. (2005). Determination of Total Monomeric Anthocyanin Pigment Content of Fruit Juices, Beverages, Natural Colorants, and Wines by the pH Differential Method: Collaborative Study. J. AOAC Int..

[60] Broadhurst R.B., Jones W.T. (1978). Analysis of Condensed Tannins Using Acidified Vanillin. J. Sci. Food Agric..

[61] Apak R., Güçlü K., Ozyürek M., Karademir S.E. (2004). Novel Total Antioxidant Capacity Index for Dietary Polyphenols and Vitamins C and E, Using Their Cupric Ion Reducing Capability in the Presence of Neocuproine: CUPRAC Method. J. Agric. Food Chem..

[62] Bobo-García G., Davidov-Pardo G., Arroqui C., Vírseda P., Marín-Arroyo M.R., Navarro M. (2015). Intra-laboratory Validation of Microplate Methods for Total Phenolic Content and Antioxidant Activity on Polyphenolic Extracts, and Comparison with Conventional Spectrophotometric Methods. J. Sci. Food Agric..

[63] Re R., Pellegrini N., Proteggente A., Pannala A., Yang M., Rice-Evans C. (1999). Antioxidant Activity Applying an Improved ABTS Radical Cation Decolorization Assay. Free Radic. Biol. Med..

[64] Sharma R., Rajput Y., Poonam, Dogra G., Tomar S. (2009). Estimation of Sugars in Milk by HPLC and Its Application in Detection of Adulteration of Milk with Soymilk. Int. J. Dairy Technol..

[65] AOAC (2005). Official Methods of Analysis.

[66] Zhou J., Qi Y., Ritho J., Zhang Y., Zheng X., Wu L., Li Y., Sun L. (2015). Flavonoid Glycosides as Floral Origin Markers to Discriminate of Unifloral Bee Pollen by LC–MS/MS. Food Control.

[67] Oboh G., Ademiluyi A.O., Akinyemi A.J., Henle T., Saliu J.A., Schwarzenbolz U. (2012). Inhibitory Effect of Polyphenol-rich Extracts of Jute Leaf (*Corchorus olitorius*) on Key Enzyme Linked to Type 2 Diabetes (α-Amylase and α-Glucosidase) and Hypertension (Angiotensin I Converting) In Vitro. J. Funct. Food..

[68] Tao Y., Zhang Y., Cheng Y., Wang Y. (2013). Rapid Screening and Identification of α-Glucosidase Inhibitors from Mulberry Leaves Using Enzyme-immobilized Magnetic Beads Coupled with HPLC/MS and NMR. Biomed. Chromatogr..

[69] Conforti F., Perri V., Menichini F., Marrelli M., Uzunov D., Statti G.A., Menichini F. (2012). Wild Mediterranean Dietary Plants as Inhibitors of Pancreatic Lipase. Phytother. Res..

[70] Mata A.T., Proença C., Ferreira A.R., Serralheiro M.L.M., Nogueira J.M.F., Araújo M.E.M. (2007). Antioxidant and Antiacetylcholinesterase Activities of Five Plants Used as Portuguese Food Spices. Food Chem..

[71] Myers R.W., Lee R.T., Lee Y.C., Thomas G.H., Reynolds L.W., Uchida Y. (1980). The Synthesis of 4-Methylumbelliferyl Alpha-ketoside of N-acetylneuraminic Acid and Its Use in a Fluorometric Assay for Neuraminidase. Anal. Biochem..

[72] Shalaby S.M., Zakora M., Otte J. (2006). Performance of Two Commonly Used Angiotensin-converting Enzyme Inhibition Assays Using FA-PGG and HHL as Substrates. J. Dairy Res..

[73] Hanif M., Shoaib K., Saleem M., Hasan Rama N., Zaib S., Iqbal J. (2012). Synthesis, Urease Inhibition, Antioxidant, Antibacterial, and Molecular Docking Studies of 1,3,4-Oxadiazole Derivatives. ISRN Pharmacol..

[74] Ribeiro J.K., Cunha D.D., Fook J.M., Sales M.P. (2010). New Properties of the Soybean Trypsin Inhibitor: Inhibition of Human Neutrophil Elastase and Its Effect on Acute Pulmonary Injury. Eur. J. Pharmacol..

[75] Hwang J.H., Lee B.M. (2007). Inhibitory Effects of Plant Extracts on Tyrosinase, L-DOPA Oxidation, and Melanin Synthesis. J. Toxicol. Environ. Health A.

[76] Kohn J., Wilchek M. (1978). A Colorimetric Method for Monitoring Activation of Sepharose by Cyanogen Bromide. Biochem. Biophys. Res. Commun..

[77] Holmgren A. (1977). Bovine Thioredoxin System. Purification of Thioredoxin Reductase from Calf Liver and Thymus and Studies of Its Function in Disulfide Reduction. J. Biol. Chem..

[78] Blum U., Schwedt G. (1998). Inhibition Behavior of Acid Phosphatase, Phosphodiesterase I and Adenosine Deaminase as Tools for Trace Metal Analysis and Speciation. Anal. Chim. Acta.

[79] Escriche I., Juan-Borrás M., Visquert M., Valiente J.M. (2023). An Overview of the Challenges When Analysing Pollen for Monofloral Honey Classification. Food Control.

[80] Kostić A.Ž., Barać M.B., Stanojević S.P., Milojković-Opsenica D.M., Tešić Ž.L., Šikoparija B., Radišić P., Prentović M., Pešić M.B. (2015). Physicochemical Composition and Techno-functional Properties of Bee Pollen Collected in Serbia. LWT—Food Sci. Technol..

[81] Ćirić J., Haneklaus N., Rajić S., Baltić T., Lazić I.B., Đorđević V. (2022). Chemical Composition of Bee Bread (Perga), a Functional Food: A Review. J. Trace Elem. Min..

[82] Thakur M., Nanda V. (2020). Composition and Functionality of Bee Pollen: A Review. Trends Food Sci. Technol..

[83] Shahali Y., Kler S., Revets D., Planchon S., Leclercq C.C., Renaut J., Shoormasti R.S., Pourpak Z., Ollert M., Hilger C. (2024). Proteome Analysis of Propolis Deciphering the Origin and Function of Its Proteins. J. Food Composit. Anal..

[84] Nemo R., Bacha K. (2021). Microbial Quality, Physicochemical Characteristics, Proximate Analysis, and Antimicrobial Activities of Honey from Anfilo District. Food Biosci..

[85] FAO (2003). Food Energy—Methods of Analysis and Conversion Factors.

[86] Leon K., Mery D., Pedreschi F., Leon J. (2006). Color Measurement in *L∗ a∗ b∗* Units from RGB Digital Images. Food Res. Int..

[87] Végh R., Sipiczki G., Csóka M. (2023). Investigating the Antioxidant and Color Properties of Bee Pollens of Various Plant Sources. Chem. Biodivers..

[88] Pauliuc D., Dranca F., Oroian M. (2020). Antioxidant Activity, Total Phenolic Content, Individual Phenolics and Physicochemical Parameters Suitability for Romanian Honey Authentication. Foods.

[89] Starowicz M., Hanus P., Lamparski G., Sawicki T. (2021). Characterizing the Volatile and Sensory Profiles, and Sugar Content of Beeswax, Beebread, Bee Pollen, and Honey. Molecules.

[90] Amorati R., Valgimigli L. (2015). Advantages and Limitations of Common Testing Methods for Antioxidants. Free Radic. Res..

[91] Kostić A.Ž., Milinčić D.D., Stanisavljević N.S., Gašić U.M., Lević S., Kojić M.O., Tešić Ž.L., Nedović V., Barać M.B., Pešić M.B. (2021). Polyphenol Bioaccessibility and Antioxidant Properties of In Vitro Digested Spray-dried Thermally-treated Skimmed Goat Milk Enriched with Pollen. Food Chem..

[92] Kasote D., Bankova V., Viljoen A.M. (2022). Propolis: Chemical Diversity and Challenges in Quality Control. Phytochem. Rev..

[93] Ali H., Abu Bakar M., Majid M., Muhammad N., Lim S. (2020). In vitro Anti-diabetic Activity of Stingless Bee Honey from Different Botanical Origins. Food Res..

[94] Keskin M., Özkök A. (2020). α-Amylase Inhibition Properties of Bee Pollen and Bee Bread (Perga). Hacettepe J. Biol. Chem..

[95] Rahmawati O., Pratami D.K., Raffiudin R., Sahlan M. (2019). Alpha-glucosidase Inhibitory Activity of Stingless Bee Honey from *Tetragonula biroi* and *Tetragonula laeviceps*. AIP Conf. Proc..

[96] Uddin S., Brooks P.R., Tran T.D. (2022). Chemical Characterization, α-Glucosidase, α-Amylase and Lipase Inhibitory Properties of the Australian Honey Bee Propolis. Foods.

[97] Baltas N., Yildiz O., Kolayli S. (2016). Inhibition Properties of Propolis Extracts to Some Clinically Important Enzymes. J. Enzym. Inhib. Med. Chem..

[98] Sato A., Sunayama J., Okada M., Watanabe E., Seino S., Shibuya K., Suzuki K., Narita Y., Shibui S., Kayama T. (2012). Glioma-Initiating Cell Elimination by Metformin Activation of FOXO3 via AMPK. Stem Cells Transl. Med..

[99] Nagai T., Nagashima T., Suzuki N., Inoue R. (2005). Antioxidant Activity and Angiotensin I-Converting Enzyme Inhibition by Enzymatic Hydrolysates from Bee Bread. Z. Naturforsch. C.

[100] Malone L.A., Giacon H.A., Burgess E.P.J., Maxwell J.Z., Christeller J.T., Laing W.A. (1995). Toxicity of Trypsin Endopeptidase Inhibitors to Honey Bees (Hymenoptera: Apidae). J. Econ. Entomol..

[101] Sagili R.R., Pankiw T., Zhu-Salzman K. (2005). Effects of Soybean Trypsin Inhibitor on Hypopharyngeal Gland Protein Content, Total Midgut Protease Activity and Survival of the Honey Bee (*Apis mellifera* L.). J. Insect Physiol..

[102] Di Petrillo A., Santos-Buelga C., Era B., González-Paramás A.M., Tuberoso C.I.G., Medda R., Pintus F., Fais A. (2018). Sardinian Honeys as Sources of Xanthine Oxidase and Tyrosinase Inhibitors. Food Sci. Biotechnol..

[103] Sahin H., Aliyazicioglu R., Yildiz O., Kolayli S., Innocenti A., Supuran C.T. (2011). Honey, Pollen, and Propolis Extracts Show Potent Inhibitory Activity Against the Zinc Metalloenzyme Carbonic Anhydrase. J. Enzym. Inhib. Med. Chem..

[104] Akkemik E., Akbulut G. (2018). An Investigation of Inhibition Effects of Honey, Pollen, Propolis and Royal Jelly Extracts on Thioredoxin Reductase Enzyme Activity. Sakarya Univ. J. Sci..

[105] Erguder B.I., Kilicoglu S.S., Namuslu M., Kilicoglu B., Devrim E., Kismet K., Durak I. (2008). Honey Prevents Hepatic Damage Induced by Obstruction of the Common Bile Duct. World J. Gastroenterol..

[106] Chicas-Mosier A.M., Black T.E., Hester K.P., Belzunces L.P., Abramson C.I. (2022). Honey Bee (*Apis mellifera ligustica*) Acetylcholinesterase Enzyme Activity and Aversive Conditioning Following Aluminum Trichloride Exposure. BMC Zool..

[107] Oh M.H., Houghton P.J., Whang W.K., Cho J.H. (2004). Screening of Korean Herbal Medicines Ssed to Improve Cognitive Function for Anti-cholinesterase Activity. Phytomedicine.

[108] Mustacich D., Powis G. (2000). Thioredoxin Reductase. Biochem. J..

[109] Dagsuyu E., Yanardag R. (2024). Purification and Characterization of Thioredoxin Reductase Enzyme from Commercial *Spirulina platensis* Tablets by Affinity Chromatography and Investigation of the Effects of Some Chemicals and Drugs on Enzyme Activity. Biotechnol. Appl. Biochem..

[110] Becerril-Sánchez A.L., Quintero-Salazar B., Dublán-García O., Escalona-Buendía H.B. (2021). Phenolic Compounds in Honey and Their Relationship with Antioxidant Activity, Botanical Origin, and Color. Antioxidants.

[111] Castiglioni S., Stefano M., Astolfi P., Pisani M., Carloni P. (2022). Characterisation of Bee Pollen from the Marche Region (Italy) According to the Botanical and Geographical Origin with Analysis of Antioxidant Activity and Colour, Using a Chemometric Approach. Molecules.

[112] Ecem Bayram N., Çebi N., Celik S., Gerçek Y.C., Bayram S., Tanuğur Samancı A.E., Sağdıç O., Özkök A. (2021). Turkish Royal Jelly: Amino acid, Physicochemical, Antioxidant, Multi-elemental, Antibacterial and Fingerprint Profiles by Analytical Techniques Combined with Chemometrics. J. Apic. Res..

[113] Miraldi E., Cappellucci G., Baini G., Pistone E.S., Allodi M., Costantino G., Spaggiari C., Biagi M. (2024). Chemical Markers in Italian Propolis: Chrysin, Galangin and CAPE as Indicators of Geographic Origin. Plants.

[114] Moraru D., Alexa E., Cocan I., Obiștioiu D., Radulov I., Simiz E., Berbecea A., Grozea A., Dragomirescu M., Vintilă T. (2024). Chemical Characterization and Antioxidant Activity of Apilarnil, Royal Jelly, and Propolis Collected in Banat Region, Romania. Appl. Sci..

