# Supplementary material for: Enzyme Inhibitory, Physicochemical, and Phytochemical Properties and Botanical Sources of Honey, Bee Pollen, Bee Bread, and Propolis Obtained from the Same Apiary
Source: Antioxidants (Basel). 2024 Dec 5;13(12):1483. doi: 10.3390/antiox13121483 (PMC11673488; doi:10.3390/antiox13121483)
Supplement: Supplementary file 1 [file antioxidants-13-01483-s001.zip › antioxidants-3307814-supplementary.pdf]

Table S1: The quantitation and confirm ions of phenolic compound standards.

Peak Number	Component Name	Retention Time (min)	Quantitation Ion	Confirm Ion 1	Confirm Ion 2
1	Gallic acid	2.11	124.88	96.88	78.88
2	Protocatechuic acid	2.24	108.88	90.88	80.88
3	Neochlorogenic acid	4.60	191.00	178.92	135.00
4	Chlorogenic acid	4.61	190.88	160.88	126.88
5	*trans*-caffeic acid	5.17	134.88	106.97	88.88
6	Catechol	7.56	107.97	80.88	90.88
7	*p*-coumaric acid	7.97	118.97	116.97	92.88
8	trans-ferulic acid	9.16	133.88	177.88	148.97
9	(-)-Epicatechin	9.17	244.92	205	202.92
10	Catechin	9.21	138.92	122.92	165.00
11	Epicatechin gallate	10.12	168.92	244.92	289.00
12	Rutin	10.33	299.88	270.88	254.88
13	Quercetin	10.42	150.88	272.88	178.88
14	Hyperoside	10.45	300.92	270.92	299.92
15	Quercitrin	11.08	300.88	299.88	270.88
16	Hesperidin	11.08	300.97	324.88	285.88
17	Myricetin	11.36	178.88	150.88	136.88
18	trans-cinnamic acid	12.27	102.97	61.88	76.88
19	Luteolin	12.52	132.88	174.88	150.88
20	Kaempherol	12.53	238.88	186.88	184.88
21	Naringenin	13.07	150.88	176.88	118.88
22	Pinobanksin	13.27	252.88	196.88	160.88
23	Apigenin	13.3	116.88	150.88	148.88
24	Pinocembrin	15.03	212.88	170.88	150.88
25	Chrysin	15.11	142.88	208.88	144.88
26	CAPE*	15.27	134.88	178.88	160.88
27	Galangin	15.34	168.88	222.88	170.88
28	Artepillin C	16.8	255.05	243.97	199.97

*CAPE: Caffeic acid phenethyl ester
